# Assessment of Time to Hospital Encounter after an Initial Hospitalization for Heart Failure: Results from a Tertiary Medical Center

**DOI:** 10.1155/2018/6087367

**Published:** 2018-04-01

**Authors:** Nicolas W. Shammas, Ryan Kelly, Jon Lemke, Ram Niwas, Sarah Castro, Christine Beuthin, Jackie Carlson, Marti Cox, Gail A. Shammas, Terri DeClerck, Kathy Lenaghan, Sunny Arikat, Marcia Erickson

**Affiliations:** ^1^Genesis Health System, Davenport, IA, USA; ^2^Midwest Cardiovascular Research Foundation, Davenport, IA, USA

## Abstract

**Background:**

Hospital inpatient readmissions for patients admitted initially with the primary diagnosis of heart failure (HF) can be as high as 20–25% within 30 days of discharge. This, however, does not include admissions for observations or emergency department (ED) visits within the same time frame and does not show a time-dependent hospital encounter following discharge after an index admission. We present data on time-dependent hospital encounter of HF patients discharged after an index admission for a primary diagnosis of HF.

**Methods:**

The study recruited patients from 2 hospitals within the same health system. 500 consecutive admissions with the ICD diagnosis of HF were reviewed by inclusion and exclusion screening criteria. The 166 eligible remaining patients were tracked for post hospital discharge encounters consisting of hospital admissions, observation stays, and ED visits. Only those with a primary diagnosis of heart failure were included. Demographics were recorded on all patients. Days until hospital inpatient readmissions or hospital encounters were displayed in Kaplan–Meier plots.

**Results:**

A total of 166 patients met inclusion criteria (mean age 79.3 years, males 54%). For the first 90 days following the index admission, there were a total of 287 follow-up visits (1.7 per patient), 1158 total hospitalization days (2.6 per visit, 7.0 per patient, and 8.6 per 100 days at risk), and 21 deaths (12.7%). At 30 days, 25% and 52% of patients had an inpatient readmission or a hospital encounter, respectively. The median time to inpatient readmission was 117 days and to hospital encounter was 27 days.

**Conclusion:**

Time-dependent excess days in acute care (unplanned inpatient admission, outpatient observation, and ED visit) rather than 30-day hospital inpatient readmission rate is a more realistic measure of the intensity of care required for HF patients after index admission.

## 1. Introduction

Heart failure (HF) is the second leading risk factor for a cardiovascular hospital inpatient readmission for patients admitted initially with the primary diagnosis of HF. It is estimated that this readmission rate can be as high as 20–25% within 30 days of discharge, creating significant direct and indirect costs to our health care system [1–4]. In an attempt to reduce this 30-day readmission rate, the Centers for Medicare and Medicaid Services (CMS) financially penalizes hospitals with higher than expected 30-day readmission rates. These risk models developed at Yale University are based upon underlying diseases and comorbid conditions and generate risk-adjusted readmission rates that are compared to the National crude rate to determine readmission penalties [5, 6].

The 30-day readmission rate is, however, an arbitrary endpoint. Many sites that are penalized hit the National crude rate in 29 or 28 days, and many sites not penalized hit the National crude rate in 31 or 32 days. In addition, CMS includes in their models the 30-day readmissions that occur outside the institution, which cannot be tracked until approximately 9 months later with the CMS audits. Furthermore, with global bundled fees, sites have to better understand how initiatives to reduce readmissions affect readmission rates and encounter rates across time. Currently, readmission rates do not include admissions for observations or emergency department (ED) visits within the same time frame and do not show a time-dependent hospital encounter following discharge after an index admission.

We present data on time-dependent hospital encounters of HF patients discharged after an index admission for a primary diagnosis of HF at a large tertiary health care system.

## 2. Methods

This is a historical cohort study of patients treated at either Genesis Medical Center-Davenport or Genesis Medical Center-Silvis for their initial qualifying primary diagnosis of heart failure (HF). The study includes admissions between July 2010 and June 2012 with up to 180 days for additional readmissions through December 31, 2012. The primary endpoint is the time to hospital patient encounter after hospital discharge for qualifying patients with the diagnosis of HF. A hospital encounter was defined as either a hospital admission, observation status, or an emergency department (ED) visit. This study was conducted following all applicable local and federal regulations. The study was approved by the Institutional Review Board (IRB) of the Genesis Health System. A waiver of informed consent and a waiver of HIPPA authorization were obtained from the IRB. All information and data concerning subjects or their participation in this study were considered confidential. All data used in the analysis and reporting of this evaluation were used in a manner without identifiable reference to the subject.

### 2.1. Inclusion Criteria

Subjects had to meet all of the following criteria to be included in the study:

1. Patients had their initial admission with primary diagnosis of HF whether systolic or diastolic on or after July 1, 2010.
2. Patients resided within the Genesis Health System 17-county service area. They resided in the 9-county primary service area including Scott, Jackson, Clinton, Muscatine, and Des Moines counties in Iowa and Rock Island, Mercer, Henry, and Whiteside counties in Illinois, or in the secondary 8-county service area including Cedar, Louisa, Henry, and Lee counties in Iowa and Carroll, Henderson, Knox, and Warren in Illinois. Patients were also included if their reported zip code was outside this area but they were admitted from a local long-term care facility.

### 2.2. Exclusion Criteria

Subjects were not included in the study if any of the following conditions were present:

1. HF admissions with a history of HF present on admission (POA) prior to July 1, 2010
2. Hospital death at index visit
3. Patients who transferred from one acute care setting to another acute care setting
4. Patients who chose to leave against medical advice

### 2.3. Study Rules

1. All patients with an initial qualifying primary diagnosis (as coded at Genesis Medical Centers in Davenport and Silvis) of HF with an admission from July 1, 2010, through June 30, 2012, were evaluated.
2. All medical records for the index admission were retrieved as well as records of all readmissions to a hospital in the Genesis Health System prior to December 31, 2012.
3. All patients had one qualifying hospitalization but may have had multiple discharges that followed for readmission.
4. A good faith effort was used to identify dates of death from public records and Genesis records in order to censor patients from further risk of admission. The sources included Genesis medical records, the Social Security National Death Index which is updated at Genesis on a regular basis, and finally through obituaries. Genesis records are also supplemented by expired patients identified by CMS through their audits.

### 2.4. Study Sample Size

All 166 patients with a heart failure index visit meeting the screening criteria were tracked and analyzed. No patient was considered lost to follow-up unless there was evidence that they had been admitted to an acute care hospital outside of the Genesis Health System.

### 2.5. Study Variables

Demographic and hospital variables collected are listed in Tables 1 and 2.

### 2.6. Statistical Analysis

Monitoring of data was performed by a committee from the investigators who reviewed and adjudicated all reasons for readmission after index admission and reconciled any differences seen by the coding department and the reviewer as to the reason of HF admission at index (primary versus secondary). Monitoring consisted of the review of subject records, source documents, and other required documentation as needed.

Baseline data were contrasted across strata and primary and secondary service areas. Age and sex were screened to determine whether there were differences in baseline data. Analyses included both parametric and nonparametric as was appropriate for the underlying distribution. For sparse data, exact tests from the CYTEL Studio (Cytel, Cambridge, MA) were used and for others MINITAB (Minitab Inc.) was used.

Cox proportional hazards analysis was used for the primary analysis of time to readmission and time to encounter. Patients were censored at the time of their death, a disposition that was against medical advice, or a transfer from another acute care hospital. These analyses were stratified by sites performed using the TIBCO S-Plus software (TIBCO Software Co., Palo Alto, CA). Kaplan–Meier survival analyses with run tests were used to both screen the above factors and demonstrate their impact on time to readmission (MINITAB or S-Plus).

## 3. Results

A total of 500 admissions between 10/1/2011 and 9/30/2013 who had an ICD diagnosis of HF were reviewed using medical records, and all patient hospital encounters were recorded until 12/31/2013. A total of 166 patients met inclusion criteria (mean age 79.3 years, males 54%). Types of heart failure were systolic 33%, diastolic 33%, both 7%, and unspecified 27%. Exclusions were mostly accounted for by patients who had prior HF and those transferred from other institutions.  Table 1 illustrates patients' demographics.

**Figure 1:**
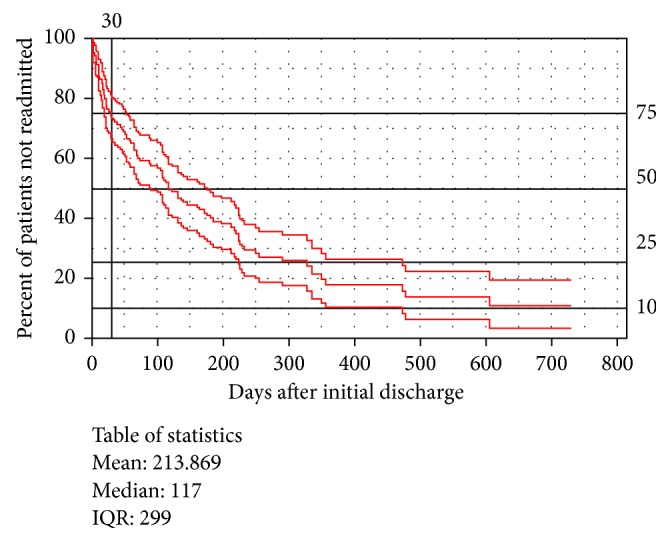
A survival curve illustrating freedom from inpatient admissions for HF after hospital discharge with the primary diagnosis of HF.

For the first 90 days following the index admission, there were a total of 287 follow-up visits (1.7 per patient), 1158 total hospitalization days (2.6 per visit, 7.0 per patient, and 8.6 per 100 days at risk), and 21 deaths (12.7%).  Figure 1 is a survival curve illustrating freedom from inpatient admissions for HF after hospital discharge with the primary diagnosis of HF.

Table 2 illustrates the predictors of encounter after hospital discharge with the primary diagnosis of HF. Among the several significant predictors, noncardiac causes such as depression and chronic obstructive pulmonary disease have the highest risk of an encounter within 30 days after an initial hospital discharge.

**Figure 2:**
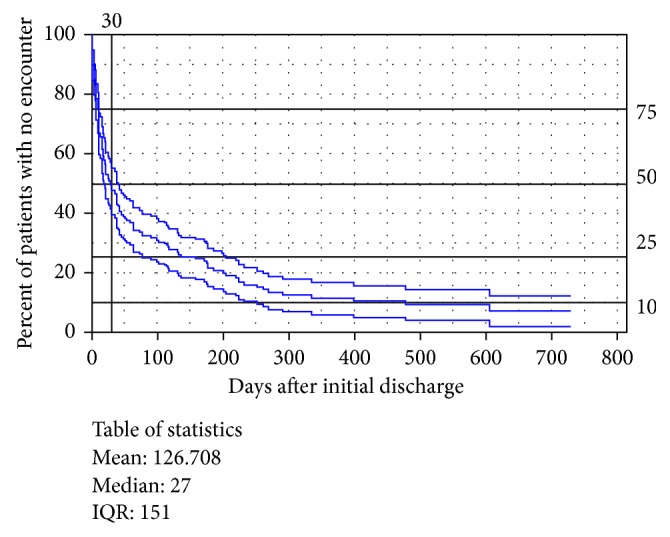
A survival curve illustrating freedom from encounters for HF after hospital discharge with the primary diagnosis of HF.

At 30 days, 25% and 52% of patients had an inpatient readmission or a hospital encounter, respectively. The median time to inpatient readmission was 117 days and to hospital encounter was 27 days.  Figure 2 is a survival curve illustrating freedom from encounters for HF after hospital discharge with the primary diagnosis of HF.

In our study, the fitted probability of 30-day inpatient readmission versus CMS probability correlated significantly (*p*=0.024).

## 4. Discussion

HF readmissions impose a health burden on the health care system costing billions of dollars in direct and indirect costs. Currently, CMS penalizes hospitals for 30-day readmission of a patient discharged with the primary diagnosis of HF. Several measures have been attempted to reduce 30-day readmission for HF with various levels of success. These measures, however, focused on hospital readmission rather than patient encounter, which also include ED visits and admission for observations. The 30-day admission or encounter rates are arbitrary time points. Hospitals may project more accurately their readmission rate after a HF discharge if a time-dependent admission rate is generated.

In this study, the 30-day readmission rate was consistent with the national average of about 25%. This correlated significantly with the CMS-fitted probability, indicating that our study's sample is a true reflection of the overall HF CMS population. The encounter rates were significantly higher than the readmission rates, indicating that patients discharged with the primary diagnosis of HF seek more health care resources than projected by the 30-day readmission rate. Therefore, any bundled global fees for readmission should take into consideration the near doubling of resource requirements than what is projected by the CMS 30-day readmission rates.

It must be noted that these models treat males and females the same and that there are no interactions found. There are several limitations, however. All patients used to build these models were at least 65 years old, and therefore, conclusions derived from these data do not apply to younger patients. Also, patients readmitted outside the health system were not captured, and consequently, the current projected readmission or encounter rates may well be underestimated. In addition, patients with prior several readmissions were not included because they potentially may create a confounding factor that would influence future readmission or encounter rates. Therefore, our data only apply to those patients with first HF hospitalization. Finally, the study is relatively small in numbers and needs to be confirmed by a larger multicenter cohort of patients.

## Figures and Tables

**Table 1:** Patients' demographics.

Mean age (years)	79.3
Sex (male)	54%
Heart failure type	
Diastolic	33%
Systolic	33%
Systolic and diastolic	7%
Unspecified	27%
Site of index visit	
Davenport	61%
Silvis	39%

**Table 2:** Predictors of encounter after initial hospital discharge.

Variable	Relative risk	*p* value
Depression	3.31	0
Chronic obstructive pulmonary disease	2.83	0
Cardiorespiratory disorder	2.53	0
Other heart diseases	2.24	0.02
Fluid disorder	1.9	0
Atherosclerosis	1.77	0
Pneumonia	1.55	0
Anemia	1.55	0
Gastrointestinal disorder	1.49	0
Drug abuse	1.46	0.01
Arrhythmia	0.77	0
Male	0.71	0
Urinary tract disorder	0.53	0.01
Asthma	0.38	0.01
Dementia	0.72	0.03
